# Smartphone apps to improve fitness and increase physical activity among young people: protocol of the Apps for IMproving FITness (AIMFIT) randomized controlled trial

**DOI:** 10.1186/s12889-015-1968-y

**Published:** 2015-07-11

**Authors:** Artur Direito, Yannan Jiang, Robyn Whittaker, Ralph Maddison

**Affiliations:** National Institute for Health Innovation, University of Auckland, 261 Morrin Road, Auckland, 1072 New Zealand

**Keywords:** Physical activity, Fitness, Exercise adolescent, Health promotion, mHealth, Smartphone

## Abstract

**Background:**

Physical activity is a modifiable behavior related to many preventable non-communicable diseases. There is an age-related decline in physical activity levels in young people, which tracks into adulthood. Common interactive technologies such as smartphones, particularly employing immersive features, may enhance the appeal and delivery of interventions to increase levels of physical activity in young people. The primary aim of the Apps for IMproving FITness (AIMFIT) trial is to evaluate the effectiveness of two popular “off-the-shelf” smartphone apps for improving cardiorespiratory fitness in young people.

**Methods/Design:**

A three-arm, parallel, randomized controlled trial will be conducted in Auckland, New Zealand. Fifty-one eligible young people aged 14–17 years will be randomized to one of three conditions: 1) use of an immersive smartphone app, 2) use of a non-immersive app, or 3) usual behavior (control). Both smartphone apps consist of an eight-week training program designed to improve fitness and ability to run 5 km, however, the immersive app features a game-themed design and adds a narrative. Data are collected at baseline and 8 weeks. The primary outcome is cardiorespiratory fitness, assessed as time to complete the one mile run/walk test at 8 weeks. Secondary outcomes are physical activity levels, self-efficacy, enjoyment, psychological need satisfaction, and acceptability and usability of the apps. Analysis using intention to treat principles will be performed using regression models.

**Discussion:**

Despite the proliferation of commercially available smartphone applications, there is a dearth of empirical evidence to support their effectiveness on the targeted health behavior. This pragmatic study will determine the effectiveness of two popular “off-the-shelf” apps as a stand-alone instrument for improving fitness and physical activity among young people. Adherence to app use will not be closely controlled; however, random allocation of participants, a heterogeneous group, and data analysis using intention to treat principles provide internal and external validity to the study. The primary outcome will be objectively assessed with a valid and reliable field-based test, as well as the secondary outcome of physical activity, via accelerometry. If effective, such applications could be used alongside existing interventions to promote fitness and physical activity in this population.

**Trial Registration:**

Australian New Zealand Clinical Trials Registry: ACTRN12613001030763. Registered 16 September 2013.

**Electronic supplementary material:**

The online version of this article (doi:10.1186/s12889-015-1968-y) contains supplementary material, which is available to authorized users.

## Background

Participation in regular physical activity has been associated with better cardiometabolic indices [1–3], skeletal health [4], and cognitive and academic performance [5, 6] in young people (i.e. children and adolescents aged 7–18 years). Conversely, doing little or no physical activity has been related to poor health outcomes [7] and decreased quality of life in adulthood [8], resulting in extended medical care and associated costs [9, 10]. There is an age-related decline in physical activity levels among children aged nine years and older, with the lowest levels among adolescents (particularly females) [11–14]. This decline is important because physical activity patterns have been shown to track from adolescence into adulthood [15–17]. Increasing physical activity in young people therefore is an important public health and research priority [18].

Despite the importance of regular physical activity, young people live in an environment with increased barriers to physical activity and increased options for sedentary leisure activities [19]. Interventions targeting young people to date have met with mixed success regardless of context of delivery (e.g. home-family based, school based). Moreover, they tend to have limited reach and are resource intensive to deliver because they often involve multicomponent interventions (e.g. educational, environmental, policy) and different settings (school, school plus community, family, primary care) [20, 21]. Given their ubiquitous use by young people, mobile phones may offer a viable opportunity to reach this population and deliver interventions aimed at promoting health behaviors, including physical activity. Benefits of mobile health (mHealth) delivery approaches over traditional face-to-face methods are they can be personalized according to participant needs, and intervention content can be provided anywhere/at any time, making them potentially more accessible, scalable, and cost-effective [22, 23].

mHealth systems can be used to make health information and behavior change more appealing and entertaining through gaming [23]. There is scarce evidence on the effectiveness of health video games played on mobile devices, however, video games played on traditional systems have been shown to promote positive health-related outcomes [24, 25]. Adding physical activity to video gaming has been proposed as a strategy to increase physical activity during leisure time [26–28], with greater enjoyment associated with increased energy expenditure during play of active video games [29, 30]. Importantly, engagement, which reflects distraction from the real world, was found to predict enjoyment and, consequently, energy expenditure [29]. One important design feature to engage and sustain use of such games by making them more enjoyable is the presence of a narrative or story [31, 32]. A narrative with immersive properties—i.e. a process in which people are transported into the story—enables the suspension of disbelief, vivid personal experiences, and affection for the characters [32], all of which important in the development of intrinsic motivation [33], a key determinant of physical activity behavior [34]. An immersive narrative has been positively associated to improvements in physical activity self-efficacy in a randomized trial of a health video game to improve children’s diet and physical activity behaviors [35]. Additionally, previous studies demonstrated that a game-themed aerobic game is perceived as more enjoyable than an exercise-themed aerobic game [30, 36]. Given the target population and intervention proposed, these design features are important to consider.

Much of the evidence to date on the effectiveness of mHealth interventions has focused on the use of short-message service (SMS). This research has targeted a wide range of health conditions [37] and a number of systematic reviews support the delivery of SMS interventions to improve a range of health behaviors [38, 39]. There is growing interest in the use of SMS to promote young people’s physical activity [40], however, the utilization of smartphone technologies, including apps, is still in its infancy. Smart phone technology appears to resonate with young people. National surveys conducted in the United States (U.S.) indicate smartphone ownership among young people aged 12–17 has increased from 23 % in 2011 [41] to 37 % in 2012 [42] and is similar across ethnicity and family income. Mobile phone penetration in New Zealand mirrors these data, where household access to mobile phones increased from 74 to 84 % from 2006 to 2013 [43]. Additionally, 58 % of the U.S. young people have downloaded an application (app) to their devices [44].

In recent years there has been a proliferation in the number of ‘off-the-shelf’ apps developed to promote health behaviors. By 2013 there were more than 40 thousand apps available to the general consumer in the U.S. iTunes store health & fitness category [45]. While only approximately half were genuinely healthcare related, this still represents an immense number of apps, the majority targeting physical activity and dietary behaviors. Further, an assessment of their functionality found apps do little more than provide information [45]. Even though content analysis of apps provide preliminary insights on their potential [46, 47], there is a dearth of empirical evidence to support their effectiveness on the targeted health behavior [48, 49], including physical activity [50, 51].

**Fig. 1:**
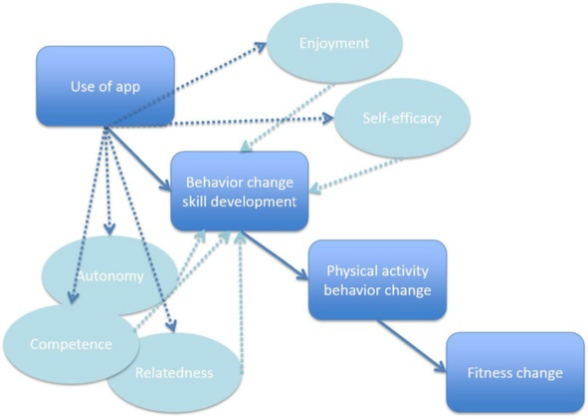
Proposed conceptual model and pathway of how use of the apps may influence possible mediators that change physical activity behavior and consequently influence fitness [86]

A proposed model for better understanding how an app intervention may influence possible mediators that change physical activity behavior and consequently improve fitness is presented in Fig. 1. It is hypothesized that the app will positively affect mediating variables, such as enjoyment, autonomy, competence, relatedness, and self-efficacy; which in turn would result in physical activity-related changes in fitness. We identified two commercially readily available apps, both designed to promote increases in walking and running, which are physical activity behaviors that contribute to improvements in fitness. Additionally, we are interested in comparing an app designed with an immersive story to an app without story since game-based stories that engage individuals can be used for health behavior change [24, 35] to model behaviors by immersing and capturing attention.

Therefore, the primary aim of the Apps for IMproving FITness (AIMFIT) trial is to evaluate the effectiveness of two popular ‘off-the-shelf’ smartphone apps for improving cardiorespiratory fitness in young people aged 14–17 years, compared to usual behavior alone (the control). Secondary goals are to determine the effect of the interventions on physical activity levels, self-efficacy, enjoyment, and psychological need satisfaction. Perceptions of usability and acceptability of the apps will also be assessed.

## Methods/design

An eight-week, three-arm parallel randomized controlled trial will be conducted in Auckland, New Zealand with a total of 51 eligible participants. The primary outcome is cardiorespiratory fitness at 8 weeks after randomization. We hypothesize that participants who receive a smartphone app will have greater cardiorespiratory fitness compared to the control group. Secondarily, we hypothesize fitness, physical activity levels, self-efficacy, enjoyment and psychological needs satisfaction will be greater in the immersive app group compared to the non-immersive app group at 8 weeks after randomization. We also expect that recruitment goals will be met, attrition will be low, and that we will be able to collect complete data on at least 80 % (41/51) of participants.

The protocol is in accord with the SPIRIT 2013 statement [52, 53], and the intervention is described according to the CONSORT-EHEALTH checklist [54]. See Additional file 1 for the complete checklist.

### Study sample and recruitment

Eligible participants are English-speaking New Zealand young people aged 14–17 years, living in Auckland, who are owners of an iPod touch® or smartphone running at least Android 2.2 or iOS 6.0, and are able to perform physical activities but do not meet the New Zealand guidelines for regular physical activity [55] (i.e. at least 60 min of moderate to vigorous physical activity each day). Exclusion criteria are a medical condition limiting their ability to exercise safely, previous use or download of the apps of interest, and inability to comply with the study protocol. Only one child per household is eligible to take part.

Several methods are used for participant recruitment including university electronic mailing lists, advertisements in local newspapers, flyers posted in community locations, and visits to high schools to present the study. Even though subgroup analyses of certain demographics or socioeconomic groups is not a goal of this study, to improve access of indigenous and ethnic minority populations to the intervention we visited schools of the greater Auckland area where a large number of Maori and Pacific reside. The criteria for selection were broad in an attempt to include a diverse sample, reduce homogeneity and improve the generalizability of the results.

Interested participants either initiate contact with the research team or their contact details are obtained from sign-up sheets delivered while visiting schools to present the study. During an initial telephone call, interested participants are screened for eligibility [56] and a study information sheet is either posted or emailed. A baseline assessment is scheduled for eligible participants within 4 weeks of the screening call. Parents/caregivers provide written informed consent for their child (14–15), while the child provides written assent. Those aged 16–17 years provide their own written informed consent. Participants will receive a NZ $30 gift card to a local shopping center as an incentive for participation and encourage completion of study measures. Incentives are not conditional on usage of the app. Moreover, participants will be told both apps will be provided (i.e. free of charge) at study termination, which is particularly important to meet the expectations of using physical-activity related apps of those in the control group in order to avoid high dropout rates. As adherence to the intervention is one of the outcomes of interest, no reinforcement strategies will be used to influence compliance.

### Outcome assessments

**Fig. 2:**
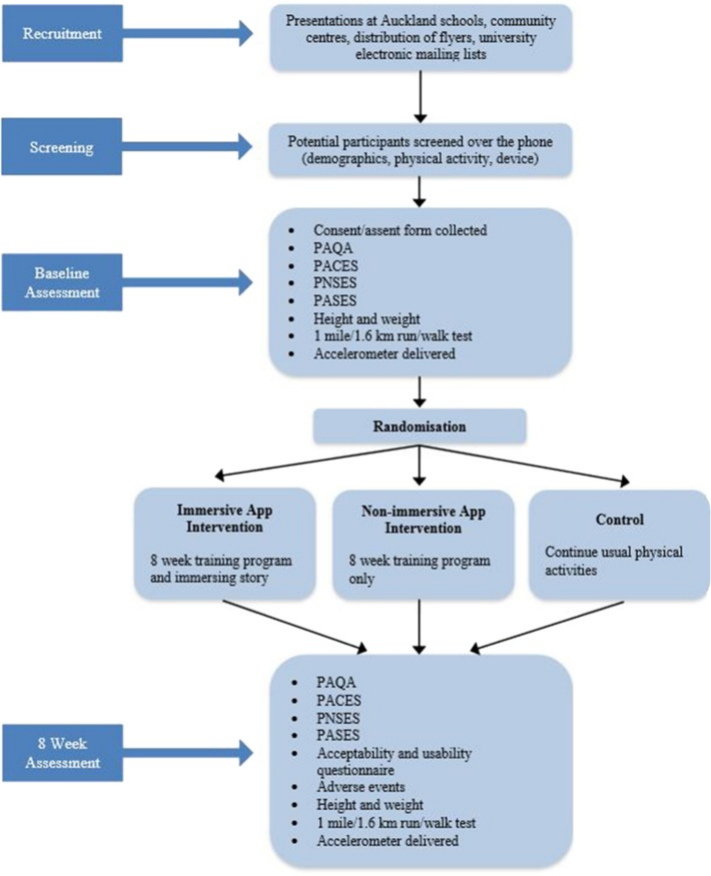
Flow diagram of the study. Legend: PAQA-Physical Activity Questionnaire for Adolescents, PACES-Physical Activity Enjoyment Scale, PNSES-Psychological Need Satisfaction in Exercise Scale, PASES-Physical Activity Self-Efficacy Scale

Assessments are conducted at baseline and 8-week post randomization (see Fig. 2) at the University of Auckland clinics and sport grounds (duration 45 to 60 min, of which 10 to 15 min to complete self-reported measures). Baseline assessments involve an explanation of study procedures, signed consent and collection of participant-reported secondary outcomes, followed by physical measurements (height and weight) and a field test of cardiorespiratory fitness. Participants are then asked to wear an accelerometer for seven consecutive days. Randomization takes place at a subsequent visit to the participants’ home once baseline accelerometer data collection is completed.

### Ethical approval

Ethical approval was obtained from the University of Auckland Human Participants Ethics Committee (10054/2013). The study was registered at the Australian New Zealand Clinical Trials Registry before commencement (ACTRN12613001030763). Adverse events are collected at each visit or voluntarily reported by contacting the investigator.

### Sample size

A total sample of 51 participants (17 per group) will provide 80 % power at 5 % level of significance (two-sided) to detect a minimal difference of 17 s in time to complete the one mile run/walk test at 8-week post randomization between the treatment groups. This has assumed a standard deviation of 15 s on the primary outcome, with adjustment for multiple comparisons. Based on normative data [57] for this population, the calculation assumes a mean baseline value of 10-min to complete the one mile/1.6 Km run/walk test. An improvement in the completion time of 78 s for this distance corresponds to a shift from the 25^th^ to the 50^th^ percentile in terms of cardiorespiratory fitness, which is considered a meaningful impact towards a more favorable cardiovascular profile.

### Randomization and blinding

Following the baseline measurement, participants are randomized at a 1:1:1 ratio to one of three conditions. The randomization sequence was generated by the study statistician using a computer program in variable blocks (3 and 6), stratified by sex [58]. Allocation was concealed in consecutively numbered, opaque sealed envelopes. Given the nature of the intervention, participants are aware of the group to which they have been allocated. The outcome assessor will not be blinded to the treatment allocation, however, the assessment of cardiorespiratory fitness and physical activity levels utilize objective measures.

### Description of interventions

Participants randomized to the intervention groups receive either a non-immersive or immersive app (see description below), which is downloaded on their mobile device by AD. Once installed, participants receive a short instruction on the features and settings of the app and are encouraged to use it three times per week. No changes will be made to the intervention or study design once recruitment begins, however, participants can choose to stop receiving the intervention and will be informed of this during the consenting process.

The choice for the interventions was based on previous work conducted by the authors, which evaluated the top-40 most downloaded apps of the health and fitness category of the New Zealand iTunes store. In this study we rated "off-the-shelf" apps for the presence or absence of behavior change techniques [46]. The two apps selected included self-regulatory behavior change techniques previously shown to be effective in changing physical activity behavior (i.e. prompt intention formation, prompt specific goal setting, prompt self-monitoring, provide feedback on performance, and prompt review of behavior goals) [59], were focused on improving fitness, and were available in both the Apple and Android stores. Further, we were interested in comparing an app with an immersive story to an app without story since game-based stories that engage individuals can be used for health behavior change [24, 35] to model behaviors by immersing and capturing attention. Lastly, these apps are readily available to consumers and are inexpensive (NZ$2.45–4.19, depending on app store).

### Non-immersive app

**Fig. 3:**
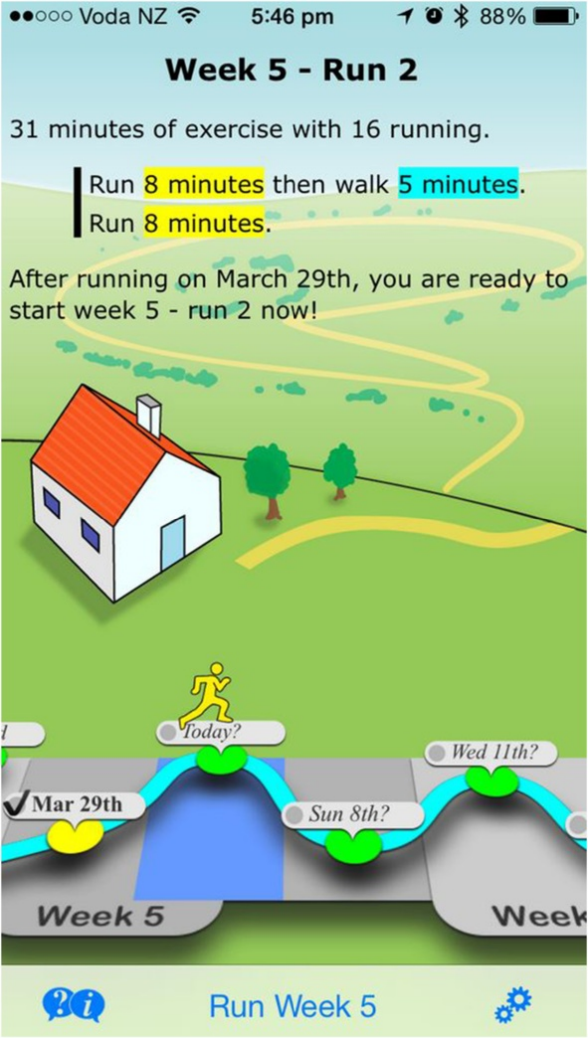
Screenshot of the “Get running” app

Participants randomized to the non-immersive app group have the “Get Running—Couch to 5 K” (Fig. 3) app installed on their smartphone/iPod touch®. This app entails a 9-week training program delivered via headphones with three workouts a week (each during 35 min approximately) to help individuals run “even if getting off the couch is a struggle” [60]. The embedded human voice coach progressively enhances the user’s fitness until they are able to complete a 5 km distance.

### Immersive app

**Fig. 4:**
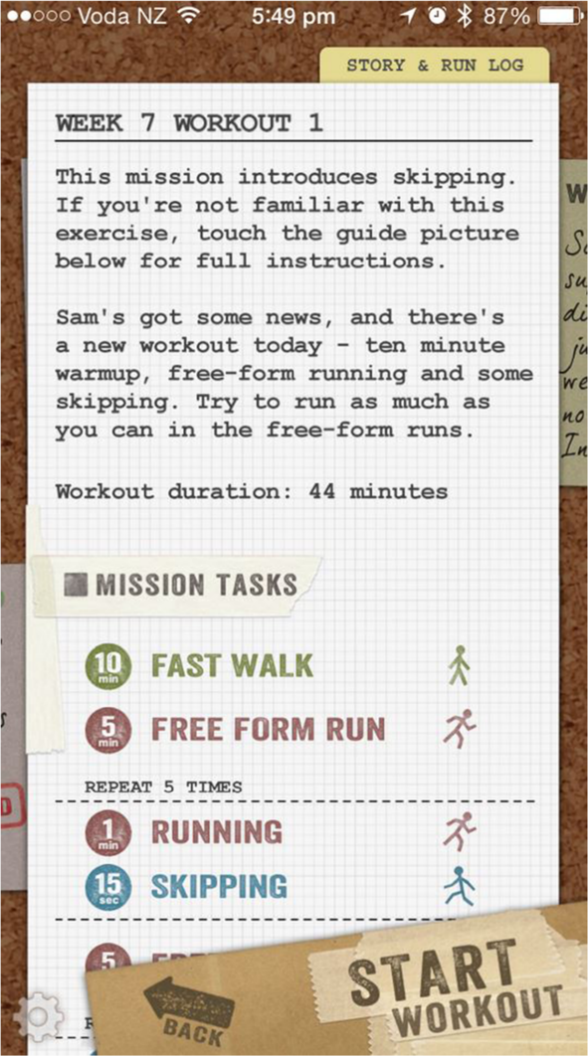
Screenshot of the “Zombies, run!” app

Participants randomized to the immersive app group have the “Zombies, Run! 5 K Training” app installed onto their device (Fig. 4). Likewise, the app consists of an 8-week training program; three workouts a week, each during 35 min approximately, that give detailed instructions via headphones about when to walk, jog, run and stretch. However, this app combines the training program with an immersing, fun story where the user needs to get fit to escape zombies. Despite its “fear themes” and “frequent/intense cartoon or fantasy violence” it is rated as suitable for an audience aged 12+ [61].

### Control (usual physical activity)

Participants randomized to the control group are asked to continue with their usual physical activities for the duration of the study. Both apps are offered to participants once they have completed the study.

Although the duration of the training program of the non-immersive app is 1-week longer than the immersive app, the programs are similar regarding their frequency and duration of sessions. All participants will be assessed 8-week post randomization.

### Outcomes

#### Primary outcome

The primary outcome is cardiorespiratory fitness, assessed with the one mile run/walk test, is a reliable and valid assessment of cardiorespiratory fitness [62, 63] that can be used to estimate oxygen consumption (VO_2_) in young people [64]. The test is used in the most important fitness test batteries for young people, such as Fitnessgram. Participants walk and/or run at their own pace until completing the distance in the shortest possible time. The total time (i.e. in minutes and seconds) to complete the course is recorded for each participant at both visits, which will be computed in seconds for subsequent analysis. Participants will complete the test individually, on an outdoor field-track on the University of Auckland campus sport grounds. Before the test, warm-up includes a walk on the track and a self-selected routine. The procedures listed in the test administration protocol will be followed [62], whereby participants will be instructed to “run the distance at the fastest pace possible” and asked to try to pace themselves in order to avoid running too fast early and then walk in the later stages. Even though the goal is to complete the distance as quickly as possible, it will be stressed that if one must walk, he/she should try a fast pace and not feel inferior. During the test, participants will be told times as they pass the start line—to assist with pacing—and encouraged with positive feedback. A list of typical encouragement sentences will be used to ensure consistency. Participants will report their rating of perceived exertion before and at the end of the assessment using the Children’s OMNI scale [65]. Following the test, participants will be free to walk around to cool-down and invited to stretch their leg muscles.

#### Secondary outcomes

The following secondary outcomes are assessed at baseline and 8 weeks.

1. Anthropometrics (body mass index) are measured using standardized practices [66]. Bodyweight is measured to the nearest 0.1 kg using a calibrated scale (Salter), while height is measured to the nearest 0.1 cm using a stadiometer (Seca). Physical measurements are taken as the average of two readings.
2. Self-reported physical activity is assessed using the Physical Activity Questionnaire for Adolescents (PAQ-A), a validated self-report seven day recall measure with good internal consistency (Cronbach alphas range from 0.72 to 0.85) and acceptable validity compared to an activity monitor (rho = 0.49) [67]. The PAQ-A items assess physical activity at school (i.e. physical education, lunchtime), after school, and at home (organized and recreational). Each statement is scored on a five-point scale with higher scores indicating higher activity levels. The final PAQ-A score is an average of eight items [68].
3. Objectively measured physical activity is assessed using the GT1M Actigraph (http://www.actigraphcorp.com), a motion sensor shown to be a valid and reliable instrument for assessing physical activity in young people [69]. Participants are asked to wear the Actigraph following each assessment for seven consecutive days. They are instructed to wear the monitor during waking hours on their mid-axilla line right hip, removing it when engaging in activities involving water (e.g. water sports, showering) and contact sports. Participants are asked to complete a daily log detailing the time the Actigraph was put on and removed.
  The GT1M model was initialized to collect data in 10-s epochs [70]. The ActiLife software (version 5) [71] is used to initialize the accelerometers and download the data following wearing periods. All accelerometer data files are exported individually as a .csv file and aggregated into a combined .csv file that is imported into SAS. During subsequent processing, data are aggregated into minute intervals. To determine valid wear time, periods of more than 60 min of consecutive zeroes and days with less than 600 min of valid records are removed before data analysis [72]. All participants with at least 3 days of valid data will be included in the analysis [73]. The average daily time spent in sedentary, light, moderate, vigorous and moderate to vigorous activities is calculated for each participant. The Evenson cut points for defining activity intensities are used: sedentary <100, light >100, moderate ≥2296, vigorous ≥4012 [74–76].
4. Perceived enjoyment of physical activity is assessed using the Physical Activity Enjoyment Scale (PACES) [77]. Participants rate their agreement with 16 statements on a five-point Likert type scale (e.g. “When I am active it gives me a strong feeling of success”). Factorial validity and gender invariance among older adolescents has been previously demonstrated [78]. Scores are summed with higher scores indicating higher level of enjoyment.
5. The Psychological Need Satisfaction in Exercise Scale (PNSES) [79] is used to measure perceived competence, autonomy and relatedness while exercising. Participants rate their agreement with 18 items on a six-point Likert type scale (e.g. “I feel free to exercise in my own way”). Six items are referenced to each of the three domains. Construct validity and gender invariance for the PNSES have been previously documented [79].
6. Self-efficacy is assessed using the Physical Activity Self-Efficacy Scale (PASES) [80]. This eight item questionnaire has well-established validity [81]. Items are scored from on a 3-point Likert-type scale (e.g. “I can be physically active most days after school”), with a higher summed score indicating higher self-efficacy.
7. Self-reported frequency of app utilization (e.g. “How many times per week did you use the app?”), acceptability (e.g. Will you continue to use the app?”), and usability (e.g. “When did you use the app?”) of the apps are assessed via an exit survey conducted with all intervention participants. A series of open-ended questions are asked to determine the features participants considered most and least acceptable, as well as the ones they used most often to support their physical activity. Objective data on app usage (i.e. automatically logged in the background while features were accessed) was not possible to collect due to the apps not being developed by the research team and not being open source (i.e., could not be modified to suit investigation purposes).

### Statistical analyses

Treatment evaluation will be performed on the principle of intention to treat. Statistical analyses will be conducted using SAS version 9.3 software (SAS Institute, Cary, NC, USA). All statistical tests will be two-sided and maintained at a 5 % significance level. Apart from accelerometry, study data will be keyed into an Excel database using data forms enabled with validation criteria. An assistant will randomly check 30 % of the keyed data for accuracy. All data will be imported into SAS for final analysis.

Baseline characteristics of all randomized participants will be presented for each group using descriptive statistics. Continuous variables will be reported as numbers of observed and missing values, mean, standard deviation, median and range. Categorical variables will be described as frequencies and percentages.

Analysis of covariance (ANCOVA) regression model will be used to evaluate the main treatment effects on the primary outcome, adjusting for its baseline measure, sex, and important baseline confounding factors. Normality of the continuous outcome data will be tested, and the goodness of model fit will be evaluated. Model-adjusted means will be estimated for each group, and their differences will be tested. A similar approach will be used for other continuous secondary outcomes. Generalized linear models will be applied to categorical outcomes as appropriate. Sensitivity analysis will be conducted on the primary outcome with missing data (if >10 %), using multiple imputations with the MCMC method [82].

**Fig. 5:**
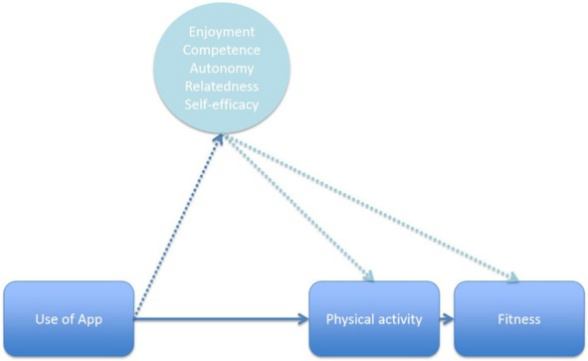
Mediation model to be tested (dotted lines indicate hypothesized mediation paths)

Psychosocial variables (i.e. perceived enjoyment, autonomy, competence, relatedness and self-efficacy) related to physical activity will be considered as potential mediators of the intervention (Fig. 5 illustrates the mediation model to be tested; dotted lines indicate hypothesized mediation paths) [83]. The recommendations of Kraemer for testing mediators of intervention effects in randomized clinical trials will be followed [83]. Data permitting, further analyses will evaluate the treatment effects on a subgroup of participants who report having adhered to the intervention.

## Discussion

The AIMFIT study aims to investigate the effectiveness of two commercially available smartphone applications to improve fitness and activity levels in young people. While there is considerable potential for enhancing the reach of behavior change interventions through the use of smartphone apps, their effectiveness as a stand-alone approach is yet to be determined. This randomized controlled trial will provide much needed data regarding the effectiveness of an off-the-shelf intervention involving minimal contact with the participant to promote fitness and physical activity in young people.

A pragmatic study design was chosen because we were interested in evaluating the effects of the apps in a real or everyday routine [84]. The AIMFIT participants use the app at locations and times of their choice where app utilization tends to occur, which will maximize the findings’ ecological validity. The intervention and its delivery are allowed to vary (e.g. different devices, different utilization of the apps), respecting the participants’ decisions, and no additional efforts will be made for its standardization. While we recognize this limitation, the present study will provide insights of the usefulness of the intervention in a real world setting where it would be implemented, thereby increasing the applicability and generalizability of the results [85]. Although frequency and duration of self-initiated use of the apps will neither be directly observed nor reported by the participants during the intervention, those in the experimental groups will self-report frequency of app use at study completion on the exit survey, which, despite prone to recall and social desirability bias, is a relevant outcome per se. Co-interventions (i.e. intervention groups access other apps) and/or contamination (i.e. control group accesses the apps under investigation) are a limitation of this design. However, and importantly, we were targeting a young population (i.e. 14–17 years) and the apps under investigation were paid, not free. While not expensive, purchasing an app on an app store requires a credit card, which young people do not typically own.

In the current study we will investigate apps readily available for the public/consumer to purchase, rather than apps designed by the research team. This approach has caveats in that we are limited to the decisions made by the app developers, such as the content of the app, duration of the training program, and other design features. The research team is unable to objectively access data on app usage (e.g. which and when features are used) as we would have had we programmed our own app. Moreover, the short duration of the chosen intervention precludes investigation of long-term effects.

The apps herein investigated were designed to promote fitness through an eight-week progressive training program based on walking, brisk walking, and running. Arguably, running may not be as easily incorporated in daily life activities as compared to walking, and therefore be less sustainable in the long-term. However, we were interested in the effectiveness of these commercially available apps and running is more likely to promote short-term effects in fitness than walking alone would. Additionally, promoting a more intense aerobic activity (i.e. running) will likely be perceived as an “exercise activity”, and intervention features that attempt to make this activity more immersive, and consequently engaging, is the purport of one of the apps under investigation. The AIMFIT study design and proposed interventions reflect these considerations and the target population. One of the strengths of the study is the objective measurement of the main outcome and of physical activity using accelerometers. Despite its shortcomings, this trial will provide valuable insight into the effects of using a non-immersive “workout context” app or an immersive “gaming context” app to improve fitness and physical activity. Moreover, secondary outcomes will be measured to determine the impact of the intervention on behavior and psychosocial processes. Physical activity levels and psychosocial variables (i.e. self-efficacy, enjoyment and psychological needs satisfaction) will be assessed to determine their potential mediating effects. The presence of mediating effects will indicate the ability of the apps to change physical activity behavior and/or psychological variables, which are underlying mechanisms through which the apps might achieve its effects on fitness.

The AIMFIT trial will also inform on important aspects such as recruitment, compliance, utilization of the intervention, retention, adverse events, and potential issues specific to the context of the research. These issues will be of interest and can provide guidance to those interested in mHealth in general and specifically to those conducting similar work.

## Conclusion

Despite the proliferation of commercially available smartphone apps, there is a dearth of empirical evidence to support their effectiveness on the targeted health behavior. The AIMFIT pragmatic randomized controlled trial will provide much needed data regarding the effectiveness of an off-the-shelf intervention involving minimal contact with the participant to promote fitness and physical activity in young people.

### Trial status

Recruitment for the trial was completed in July. Follow-up assessments were completed in September 2014 and data analysis is ongoing.

## Additional files

Additional file 1:
**SPIRIT 2013 Checklist.** The SPIRIT checklist lists items to be included in the protocol. All items are accounted for either in the manuscript or in this file.

## Abbreviations

Apps: Applications
SMS: Short messaging service
PAQ-A: Physical Activity Questionnaire for Adolescents
PACES: Physical Activity Enjoyment Scale
PNSES: Psychological Need Satisfaction in Exercise Scale
PASES: Physical Activity Self-Efficacy Scale
NZ: New Zealand
AIMFIT: Apps for IMproving FITness
