# Supplementary material for: Smartphone apps to improve fitness and increase physical activity among young people: protocol of the Apps for IMproving FITness (AIMFIT) randomized controlled trial
Source: BMC Public Health. 2015 Jul 11;15:635. doi: 10.1186/s12889-015-1968-y (PMC4702326; doi:10.1186/s12889-015-1968-y)
Supplement: Additional file 1: — SPIRIT 2013 Checklist. The SPIRIT checklist lists items to be included in the protocol. All items are accounted for either in the manuscript or in this file. [file 12889_2015_1968_MOESM1_ESM.docx]

Additional file 1. SPIRIT 2013 Checklist

| Section/Item | Item Number | Included in manuscript (Y/N) or described below |
| --- | --- | --- |
| **Administrative Information** |  |  |
| Title | 1 | Y |
| Trial registration | 2a | Y |
|  | 2b | Y |
| Protocol version | 3 | Version 4, 30/09/2013 |
| Funding | 4 | Y |
| Roles and responsibilities | 5a | Y |
|  | 5b | Y |
|  | 5c | Y |
|  | 5d | N/A, The steering committee is comprised of the study authors. |
| **Introduction** |  |  |
| Background and rationale | 6a | Y |
|  | 6b | Y |
| Objectives | 7 | Y |
| Trial design | 8 | Y |
| **Methods** |  |  |
| Participants, interventions, and outcomes | | |
| Study setting | 9 | Y |
| Eligibility criteria | 10 | Y |
| Interventions | 11a | Y |
|  | 11b | Y |
|  | 11c | Y |
|  | 11d | Y |
| Outcomes | 12 | Y |
| Participant timeline | 13 | Y |
| Sample size | 14 | Y |
| Recruitment | 15 | Y |
| Assignment of interventions |  |  |
| Allocation |  |  |
| Sequence generation | 16a | Y |
| Allocation concealment mechanism | 16b | Y |
| Implementation | 16c | Y |
| Blinding (masking) | 17a | Y |
|  | 17b | Y, The PI is not blinded. However, outcomes are assessed using objective measures. |
| Data collection, management and analysis | | |
| Data collection methods | 18a | Y, Data collection forms are stored in the trial master file. |
|  | 18b | Y |
| Data management | 19 | Y, Data will be entered by outcome assessors into a password protected secure Microsoft Excel spread-sheet. Data range checks will be in place to promote data quality. Data will be manually checked against source documents periodically throughout the study by a monitor external to the study. |
| Statistical methods | 20a | Y |
|  | 20b | Y |
|  | 20c | Y |
| Monitoring |  |  |
| Data monitoring | 21a | N/A, A DMC is not necessary as the trial is low risk. |
|  | 21b | N/A, No interim analyses are planned as the trial is low risk. |
| Harms | 22 | Y |
| Auditing | 23 | N/A |
| **Ethics and dissemination** |  |  |
| Research ethics approval | 24 | Y |
| Protocol amendments | 25 | Y, Amendments will be communicated to steering committee, ethics boards, and trial registry if necessary. |
| Consent or assent | 26a | Y |
|  | 26b | N/A |
| Confidentiality | 27 | Y, All participants are assigned a unique registration number used on data collection forms. |
| Declaration of interests | 28 | Y |
| Access to data | 29 | Y |
| Ancillary and post-trial care | 30 | Y, Described in consent form and participant information sheets. |
| Dissemination policy | 31a | Y, |
|  | 31b | Y, No professional writers will be used. |
|  | 31c | Y, There are no plans to make the data set public. |
| **Appendices** |  |  |
| Informed consent materials | 32 | Y, Consent Forms and Participant Information Sheets stored in the trial master file. |
| Biological specimens | 33 | N/A |
